# Systemic Therapy in Metastatic or Unresectable Well-Differentiated/Dedifferentiated Liposarcoma

**DOI:** 10.3389/fonc.2017.00292

**Published:** 2017-11-30

**Authors:** Yevette McGovern, Charlie D. Zhou, Robin L. Jones

**Affiliations:** ^1^Royal Marsden Hospital/Institute of Cancer Research, London, United Kingdom; ^2^Royal Free London NHS Foundation Trust, London, United Kingdom

**Keywords:** sarcoma, chemotherapy, treatment outcome, phase II trials, phase III trials, preclinical research

## Abstract

Liposarcoma is one of the most common subtypes of soft-tissue sarcoma and consists of three main subtypes, of which well-differentiated liposarcoma and dedifferentiated liposarcoma account for 40–45%. The current mainstay of systemic treatment for patients with metastatic or unresectable disease remains doxorubicin with or without ifosfamide in the first-line setting. Recently, eribulin and trabectedin have been approved by the US Food and Drug Administration for recurrent liposarcomas and progress in molecular characterization of these tumors has opened up new and potential novel treatment targets. This review will focus on the evidence base for current treatment strategies and will also discuss potential future options.

## Introduction

Liposarcoma accounts for around 15% of the overall incidence of soft-tissue sarcomas (STSs). Well-differentiated liposarcoma (WDLS) and dedifferentiated liposarcoma (DDLS) are the most common histological subgroups of liposarcoma (1). WDLS/DDLS tumors are particularly associated with aberrations in chromosome 12q13-15 involving oncogenes including *CDK4* and *MDM2* (2). The other major subtypes include myxoid liposarcoma and pleomorphic liposarcoma.

Well-differentiated liposarcoma often present as slow growing masses in the retroperitoneum and the extremities. While pure WDLS have no propensity for metastatic spread, local recurrence is a major problem for WDLS located in the retroperitoneum (3). Furthermore, the development of DDLS is an ominous feature associated with higher risk of developing metastatic disease.

Of the other histological subtypes, myxoid liposarcoma is considered to be relatively chemosensitive, particularly to anthracyclines and trabectedin (4, 5). As a result, neoadjuvant or adjuvant chemotherapy may have a role in this disease. In pleomorphic liposarcoma, the role of systemic therapy is poorly defined; there are only a few retrospective studies that suggest a degree of chemosensitivity in the metastatic setting. Here, we focus specifically on the management of WDLS/DDLS.

Surgical resection remains the definitive management for operable WDLS/DDLS disease. The vast majority of extremity WDLS can be resected with negative margins and their clinical behavior does not warrant the use of chemotherapy in either the adjuvant or neoadjuvant setting. However, in the metastatic or unresectable setting, WDLS/DDLS are considered relatively chemotherapy resistant and there is no consensus to warrant use of systemic treatment currently in the adjuvant or neoadjuvant setting.

For patients with unresectable or metastatic WDLS and DDLS, the standard treatment consists of chemotherapy, usually with an anthracycline in the first line, perhaps in combination with ifosfamide when rapid disease control is required. However, recent studies evaluating combination treatments with monoclonal antibodies and targeted agents have the potential to completely alter the current *status quo* and it is possible that the next few years will see a significant shift in the standard management of this disease.

In this review, we aim to discuss the evidence behind the current treatment strategies and to discuss the latest novel treatment options, both possible and potential (Appendix S1 in Supplementary Material). Where possible/available, specific evidence in WDLS and DDLS will be explored.

## Conventional Cytotoxic Chemotherapy/Marine-Derived Compounds Doxorubicin/Ifosfamide

Anthracycline-based chemotherapy and doxorubicin, in particular, has been the standard first-line chemotherapy in metastatic STS for over 30 years (6, 7). Due to the rarity of STS, early clinical trials enrolled patients of diverse histological subtypes into the same studies. Early reported response rates of metastatic STS to single-agent doxorubicin were in the order of 20% associated with a median survival of approximately 8 months (8). Subsequent pooled analyses have reported comparable response rates (16–27%) and median survival (7.3–12.7 months) for single-agent doxorubicin in the context of advanced or metastatic STS (9).

In one phase II study, single-agent ifosfamide demonstrated a response rate of up to 25% [95% confidence interval (CI): 13–39%] as first-line therapy with median survival of 44–52 weeks (10). In pretreated patients, including those who had initially received single-agent doxorubicin, ifosfamide as second-line demonstrated a response rate of up to 8% (CI: 2–20%) with median survival of 36–45 weeks.

Multiple clinical trials have investigated the efficacy of combined chemotherapy schedules of doxorubicin with ifosfamide compared to doxorubicin alone. They have consistently demonstrated improvement in disease response rates but no statistically significant difference in overall survival at the expense of increased toxicity (9). These findings have been most recently reaffirmed in the EORTC 62012 phase III trial which concluded that combination therapy resulted in significantly higher response rates (26 vs 14%, *p* < 0.0006) and median progression-free survival (7.4 vs 4.6 months, *p* = 0.003) (11). However, no significant benefit was demonstrated for median overall survival (14.3 vs 12.8 months, respectively, *p* = 0.073). Combination therapies with other alkylating agents, including palifosfamide and evofosfamide, have similarly failed to demonstrate any improvements in overall survival (12–14).

On the basis of these findings, single-agent doxorubicin remains the first-line standard of care for systemic treatment of liposarcoma. Combination with ifosfamide may be considered where rapid symptomatic control due to tumor volume is favorable. However, as will be discussed later, first-line combination treatment of doxorubicin and olaratumab may now be employed as an alternative to single-agent doxorubicin depending on availability in individual countries.

Single-agent ifosfamide has also been considered in the context of second-line therapy. In a phase II clinical trial comparing two schedules of 3 weekly ifosfamide as second-line treatment in unselected STS, objective response rates and median survival was 6% and 45 weeks in patients assigned to ifosfamide 5 g/m^2^ as a 24-h infusion compared to 8% and 36 weeks, respectively, in patients assigned to ifosfamide 3 g/m^2^ given over 4 h on three consecutive days (10). Recent small cohort retrospective studies have further suggested a role for high dose continuous infusion ifosfamide specifically in liposarcoma; reporting response rates of 23 and 32% for DDLS, even in patients already pretreated with doxorubicin/ifosfamide combination therapy (15, 16).

At present, only retrospective studies have investigated the role of systemic therapy specifically in the context of WDLS/DDLS. Objective response rates of WDLS/DDLS to systemic therapy have been reported at 11% in an initial cohort of 32 cases (17) and 12% in a subsequent larger cohort of 208 cases (18). All cases that demonstrated objective responses were treated with an anthracycline-based regimen. Comparable to that of STS in general, combination therapy of doxorubicin/ifosfamide resulted in better response rates but no improvement in overall survival. Median overall survival of WDLS/DDLS treated with systemic therapy was 15 months (18).

## Trabectedin

Originally isolated from the Caribbean tunicate *Ecteinascidia turbinata*, trabectedin is thought to mediate its antineoplastic effects in STS both directly on cancerous cells and by modulating the tumor microenvironment. At the cellular level, trabectedin binds to specific selected triplet in the DNA minor groove of activated genes, thereby inhibiting transcription and inducing double strand breaks (19, 20). The inhibition of transcription is thought to occur by three synergistic biochemical pathways: blockade and degradation of RNA polymerase II, displacement of transcription factors from gene promoters, and mechanical obstruction of DNA strand separation. Trabectedin further exhibits cytotoxic activities against tumor-associated macrophages and modulates the cytokine profile of the tumor microenvironment with an associated reduction in angiogenesis (21).

A number of non-randomized phase II studies have evaluated the role of trabectedin in pretreated STS reporting response rates of 2–8% and median overall survival of 9.2–12.8 months (22–25). Despite relatively low objective response rates, a sizeable proportion of patients derived significant benefits in terms of disease control, with one study reporting disease control in 54% of trabectedin patients (24). A comparative randomized phase II trial favored the trabectedin dosing schedule of 1.5 mg/m^2^ 24-h infusion every 3 weeks over a 0.58 mg/m^2^ 3-h infusions every week for 3 weeks of a 4-week cycle in a selected cohort of leiomyosarcoma and liposarcoma, with response rates of 5.6 vs 1.6% and median overall survival of 13.9 vs 11.8 months (26).

While phase IIb and phase III studies have failed to demonstrate any evidence supporting the role of trabectedin over doxorubicin as standard in untreated STS (27, 28). A phase III study has provided evidence supporting the superiority of trabectedin over dacarbazine as an active control in pretreated leiomyosarcoma and liposarcoma with a median PFS for trabectedin vs dacarbazine of 4.2 vs 1.5 months; hazard ratio, 0.55; *p* < 0.001. There was no statistically significant difference in overall response rates (9.9 vs 6.9%, *p* = 0.33) or median overall survival (12.4 vs 12.9 months, *p* = 0.37) between trabectedin and dacarbazine, respectively, but the trabectedin arm did achieve greater rates of clinical benefit (objective response or durable stable disease; 34 vs 19%, *p* < 0.001) and prolonged median duration of stable disease (6.0 vs 4.2 months, *p* < 0.001) (29). Where stable disease is achieved with trabectedin, there is evidence to support continued clinical benefit of continued treatment beyond six cycles—median overall survival in the continuation group was 27.9 months (95% CI: 22.8–33.6) compared to 16.5 months (95% CI: 13.0–22.2) in those who discontinued trabectedin (30).

The role of trabectedin in potential combination regimens, including doxorubicin (31–33) and gemcitabine (34), remains to be defined.

## Eribulin

Eribulin is another marine-derived compound, originally isolated from the marine sponge *halichondria okadai*. It appears to exert its mechanism of action by binding to microtubule ends, driving the formation of abnormal mitotic spindles which cannot pass through the metaphase/anaphase checkpoint and thereby inducing apoptosis (35). In a phase II study, eribulin was demonstrated to have activity against an unselected population of STS; however, treatment activity was particularly notable in patients with adipocytic sarcoma as well as leiomyosarcoma (36). In 37 patients with liposarcoma (of which 24 were dedifferentiated liposarcoma), 46.9% were progression-free at 12 weeks with a median progression-free survival of 2.6 months. Two patients with dedifferentiated liposarcoma demonstrated objective responses to eribulin treatment.

Subsequently, a randomized comparative phase III study was conducted comparing eribulin to dacarbazine in advanced liposarcoma and leiomyosarcoma (37). Although there was no difference in median progression-free survival between the two arms (2.6 vs 2.6 months, *p* = 0.23), eribulin demonstrated a statistically significant improvement in overall survival (13.5 vs 11.5 months, *p* = 0.0169). Subgroup analyses suggested that liposarcoma patients benefited from eribulin over dacarbazine with median overall survival estimates of 15.6 vs 8.4 months and this benefit was observed irrespective of liposarcoma histology (18.0 vs 8.1 months, HR = 0.43 in patients with DDLS) (38); consequently, eribulin has been approved by the US Food and Drug Administration (FDA) and the European Medicines Agency (EMA) for the treatment of liposarcoma.

## Gemcitabine/Docetaxel/Dacarbazine

Other second-line systemic treatment options for STS following the failure of anthracycline-based therapies include ifosfamide (as discussed above), gemcitabine and dacarbazine monotherapies as well as combination therapies of gemcitabine/docetaxel and gemcitabine/dacarbazine. The prospective trial study populations for these therapeutic agents have been composed of histologically heterogeneous STS and, as a result, it is difficult to draw conclusions regarding the efficacy of these regimens specifically in the context of liposarcoma.

Six phase II trials have investigated the efficacy of gemcitabine monotherapy in unselected pretreated STS, reporting response rates of 3.2–27% and median overall survival of 7.2–20 months (39–44), although many of these studies were conducted on small cohorts consisting primarily of leiomyosarcoma. Combination therapy demonstrated improved response rates (16 vs 8%) and median overall survival (17.9 vs 11.5 month) at the expense of greater toxicity. The ongoing phase III GeDDiS trial is investigating gemcitabine/docetaxel combination against doxorubicin standard therapy as first-line STS treatment. Although not yet published, preliminary reports from the investigators suggest non-superiority of the combination arm despite increased toxicity. The existing body of evidence would suggest that gemcitabine-based schedules may not be particularly active in WDLS/DDLS.

Initial phase II trials reported STS response rates of 18% with dacarbazine monotherapy (45) and 4% with gemcitabine/dacarbazine doublet therapy (46). This was followed by a phase II direct comparison which demonstrated the superiority of combination gemcitabine/dacarbazine (response rates 4 vs 12%, *p* = 0.009; median overall survival 16.8 vs 8.2 months, *p* = 0.014).

## Targeted Treatments: Cyclin-Dependent Kinase 4 (CDK4) Inhibitors

Cyclin-dependent kinase 4 allows progression of the cell cycle through phosphorylation of the tumor suppressor retinoblastoma protein (47). Amplification of the *CDK4* oncogene is noted in over 90% of cases of WDLS/DDLS (48) and two phase II trials have investigated the CDK4/CDK6 inhibitor palbociclib in patients with *CDK4* amplification and advanced disease.

The results from the first phase II trial were promising with evidence of objective response in one patient (PR), a 12-week PFS rate of 66% and a median PFS of 17.9 weeks in patients with WDLS or DDLS who had received prior systemic treatment. 29 patients were treated with 200 mg of palbociclib for 14 days followed by a 7-day rest period. The most common adverse events noted were hematological, with the most reported grade 3 or 4 adverse events being neutropenia (50%), thrombocytopenia (30%), and anemia (17%) (49).

A second phase 2 trial was reported in 2016 to assess whether a new dose and schedule would result in more manageable toxic effects with similar efficacy. Sixty patients were enrolled in this non-randomized open-label study and participants received palbociclib at a dose of 125 mg once daily for 21 days of a 28-day cycle. The median PFS was 17.9 weeks with a 12-week PFS rate of 57.2%. There was one complete response. The adverse event profile was similar in terms of events seen with the most common grade 3 or events being neutropenia (36%), anemia (22%), thrombocytopenia (7%), and no occurrences of neutropenic fever (50).

Given the heterogeneous behavior seen in WDLS and DDLS, the main caution with the results of these studies is the potential that the results are biased by the more indolent behavior of WDLS in comparison to DDLS although the initial phase II study of palbociclib did require that patients have investigator determined progression of disease prior to study entry. Further studies with drugs targeting this pathway are ongoing (such as in NCT02571829).

The possibility of a combination treatment with conventional chemotherapy is also being investigated; a phase 1 study combining flavopiridol (a pan-CDK inhibitor) in combination with 60 mg/m^2^ of doxorubicin reported that 7 of the 12 evaluable patients with WDLS/DDLS had stable disease at 3 months and one patient with WDLS and DDLS had prolonged stability of 99 weeks before withdrawing consent to remain on trial (51).

## Mouse Double Minute 2 Homolog (MDM2) Antagonism

*MDM2* amplification is a further target in the treatment of WD/DD liposarcoma. Over 90% of WD/DD liposarcomas express *MDM2* amplification. *MDM2* regulates transcription and degradation of the tumor suppressor gene p53 (52, 53), and its amplification is therefore thought to reduce levels of p53 resulting in downregulation of its tumor suppressor pathway (54).

An exploratory study enrolled patients with primary or relapsed, chemotherapy-naive WDLS or DDLS eligible for surgery who were then treated with RG7112, a small molecule MDM2 antagonist, neoadjuvantly. Biomarker assessment of RG7112 on MDM2 inhibition and p53 reactivation was the primary end point of this study. 20 patients were analyzed as part of the study (11 with WDLS and 9 with DDLS), 14 patients had MDM2 amplification and 18 patients had tumors which were p53 wild type. In most patients, the biomarker response was suggestive of the drug working *via* the planned molecular target with restoration of p53 and downstream p21 expression, a reduction in Ki67-expressing cells and an increase in the amount of apoptotic cells (although this was not significant). One patient experienced a partial response and 14 patients had stable disease (55).

Further studies into MDM2 inhibition are ongoing including a completed phase 1b study looking at MDM2 inhibition in combination with doxorubicin in STS patients (NCT01605526) from which results are awaited.

## Others: Tyrosine Kinase Receptor Inhibitors

Inhibition of angiogenesis pathways has produced therapeutic benefit in a number of cancer types. There is a growing body of evidence that biomolecular markers of angiogenesis in sarcoma correlate clinically with advanced disease and worsened prognosis (56). Several multi-target tyrosine kinase inhibitors acting on angiogenic pathways have been investigated in phase II trials in the context of liposarcoma.

In a small cohort of non-selected STS, sunitinib treatment demonstrated a median progression-free survival of 3.9 months and a median overall survival of 18.6 months in patients with liposarcoma (*n* = 17) (57). Median progression-free survival of 2 months and median overall survival of 15 months has similarly been reported for sorafenib in LS (*n* = 10) (58). However, due to lack of response according to RECIST criteria, neither of these inhibitors has progressed for further evaluation in clinical trials.

Accrual of adipocytic sarcoma in a phase II trial of pazopanib was discontinued after completion of the first step due to disappointing results at the primary endpoint—with a progression-free rate at 12 weeks of just 26% according to RECIST criteria (59). Despite this, outcomes for non-adipocytic STS subtypes were promising and the subsequent phase III PALETTE trial demonstrated improved progression-free survival comparing pazopanib to placebo in STS excluding liposarcoma (4.6 vs 1.6 months, *p* < 0.0001) (60).

There continues to be ongoing investigation into pazopanib and other tyrosine kinase inhibitors in liposarcoma—the results of which will better inform the therapeutic potential of these agents. Preliminary results from the NCT01506596 phase II study reports 12-week progression-free rate of 68.3%, median progression-free survival of 4.4 months, and median overall survival of 12.6 months of pazopanib in high- or intermediate-grade LS (*n* = 41) (61). Preclinical studies have demonstrated that tyrosine kinase receptors are constitutively activated in WDLS/DDLS and that selective inhibition of these pathways inhibits proliferation of these cell lines *in vitro* (62). As a result, further phase II studies are ongoing investigating novel inhibitors specifically in WDLS and DDLS subtypes (63).

## Olaratumab

Olaratumab is a recombinant human immunoglobulin G subclass 1 (IgG1) monoclonal antibody that specifically binds PDGFRα, blocking PDGF-AA, PDGF-BB, and PDGF-CC binding and receptor activation (64).

A randomized phase 1b/phase II study assessing the combination of doxorubicin and olaratumab vs doxorubicin alone in patients with locally advanced or metastatic STS who had not received prior anthracycline treatment was published in 2016. 133 patients were randomized to receive olaratumab plus doxorubicin or doxorubicin alone, this included a subgroup of 23 patients with liposarcoma in the phase II portion (8 in the combination cohort and 15 in the doxorubicin alone arm). The results showed a median PFS of 6.6 (95% CI, 4.1–8.3) and 4.1 months (2.8–5.4), a median OS of 26.5 (20.9–31.7) and 14.7 months (9.2–17.1), and an objective RR of 18.2% (9.8–29.6) and 11.9% (5.3–22.2), respectively (65).

Although the results regarding overall survival are striking, there appears to be a mismatch with the more modest PFS findings. This apparent discrepancy requires further evaluation and the precise mechanism of action of olaratumab in sarcoma remains unidentified. There is an ongoing pre-operative trial collecting tissue sample before and after olaratumab therapy to better define its mechanism of action in sarcoma. The results of a randomized phase III trial comparing doxorubicin plus olaratumab vs doxorubicin plus placebo are eagerly awaited and will help to define the role of this agent in advanced STS (NCT02451943). Nevertheless, the combination has been granted accelerated and conditional approval by the FDA and EMA.

## Aldoxorubicin

Aldoxorubicin is a novel albumin-binding prodrug of doxorubicin. A randomized phase 2b clinical trial published in 2015 showed statistically significant improvement in median PFS (5.6 vs 2.7 months) favoring aldoxorubicin over doxorubicin. There was no statistically significant improvement in overall survival. This study treated 123 advanced soft-tissue patients with first-line aldoxorubicin or doxorubicin. There were 19 patients with a diagnosis of liposarcoma included in this study but further conclusion based on subtype is not possible (66).

A recent abstract presented at ASCO 2017 from a phase III study of aldoxorubicin vs investigator’s choice (IC) showed benefit in patients with “L-Sarcomas”; patients with liposarcomas and leiomyosarcoma. 433 patients were enrolled in this study with 15% of the patients having a diagnosis of liposarcoma. In the presented results, patients with liposarcomas and leiomyosarcomas were grouped together and accounted for 57.5% of the enrolled total. The IC drugs included dacarbazine, doxorubicin, pazopanib, ifosfamide, and gemcitabine/docetaxel. Prior doxorubicin therapy was not an exclusion criterion. There was a statistically significant increased median PFS of 5.32 months in those receiving aldoxorubicin vs 2.96 months in those who received IC (67).

These data are not yet mature and the precise role of this agent for patients with WDLS and DDLS remains to be defined.

## Immune Checkpoint Inhibitors

While immunotherapy is not yet standard treatment in STS generally, there is considerable interest in its role as a treatment option of STS and it is an area of active research with trials currently ongoing.

Specific to WDLS and DDLS; a tissue-based study has looked at the immunogenicity of WDLS and DDLS by exploring the tumor microenvironment of these tumors. Tumor infiltrating lymphocytes were isolated from all eight resected retroperitoneal liposarcoma included in the study. This included five WDLS tumors and 3 DDLS tumors (68). Another recent tissue-based study has also examined WDLS and DDLS tumors, in addition to other STS tumors, to assess the immune phenotype of these subtypes using multiple techniques including NanoString gene expression analysis and analysis of PD-1 and PD-L1 expression (69). Overall these findings are suggestive of a naturally occurring immune response and within these tumors and they are a tempting target for immune checkpoint inhibition.

This is a rapidly evolving field and a recent abstract from the SARC028 trial is suggestive of a response in patients with UPS and DDLS; with 2 of 10 DDLS patients having a partial response with a median follow-up period across all STS patients of 14.5 months. This was a phase II study looking at the overall response rate of pembrolizumab in pretreated patients with advanced sarcoma (bone and soft-tissue) as its primary endpoint (70).

## Nuclear Export Inhibitors

A phase 1b study of the first in class nuclear export inhibitor selinexor showed some promise in STS patients. This agent is a small molecule indirect inhibitor against Exportin 1 (XPO1) which is involved in the movement of cargo proteins from the nucleus to the cytoplasm. These proteins include tumor suppressor proteins which can be inactivated through nuclear exclusion in the presence of XPO1 overactivity.

Although there were no patients who achieved objective response of the 52 evaluated, the patients with DDLS in particular showed some potentially promising findings with 6 (40%) of 15 patients showing a reduction in target lesion size from baseline, and 7 (47%) of 15 patients showing SD for 4 months or longer.

Across all dosing cohorts, the most common all grade adverse effects noted included nausea, vomiting, and diarrhea as well as fatigue, hematological toxicity, and hyponatremia. Dose escalation did not appear to correlate with a higher grade of adverse events in most cases (71).

Based on the findings from this study, a phase2/3 placebo-controlled study of selinexor is underway in patients with advanced dedifferentiated liposarcoma (NCT0260646).

## Discussion/Conclusion

Well- and dedifferentiated liposarcoma remain challenging diseases to treat. The mainstay of management is surgical resection for localized disease. Historically, the options for patients with advanced disease have been limited. The response rate and median PFS with anthracycline-based schedules are disappointing. However, recently both trabectedin and eribulin have been approved for advanced liposarcoma. Furthermore, olaratumab (in combination with doxorubicin) has been granted breakthrough designation by the FDA.

A number of promising agents are currently being evaluated in advanced WDLS and DDLS including the phase 2/3 study into selinexor in DDLS. CDK4 and MDM2 inhibitors are ongoing possibilities, particularly as potential combination therapies with conventional chemotherapy.

Immunotherapy with checkpoint inhibition is a rapidly evolving area in the story of systemic therapy for liposarcoma and recent early reports are very encouraging, particularly in the case of undifferentiated pleomorphic sarcoma but also dedifferentiated liposarcoma.

There are now a number of systemic agents available for patients with metastatic or unresectable WDLS/DDLS. The optimal treatment options for each individual patient will depend on a number of factors including extent of disease, performance status, comorbid conditions, and patient symptoms. Any potential toxicities should be outlined in detail and the relative advantages and disadvantages of treatment options should be weighed up on a case-by-case basis. For patients with solitary or oligoprogressive disease, radiation and ablation techniques may be considered if feasible. In patients with poor performance statuses and multiple comorbidities, best supportive care is an entirely reasonable approach. Unilateral nephrectomy is frequently indicated in the surgical management of retroperitoneal liposarcomas (72). In such populations, the use of ifosfamide should be very carefully considered. Similarly, the cumulative cardiotoxicity of doxorubicin needs to be taken into account in patients with concurrent cardiovascular disease.

The recommendation regarding first-line systemic therapy is dependent on the goals of therapy. In rapidly progressive symptomatic disease or in patients with tumors that could potentially be down staged for surgical resection, the combination of doxorubicin and ifosfamide could be considered in the context of comorbidities. In the palliative setting, doxorubicin and olaratumab may be considered. As discussed previously, olaratumab has been approved in a number of countries following the results of a randomized phase II trial (65); however, funding is not available in all countries.

Second-line therapy and beyond again depends on comorbidities and to a certain extent patient preference. There are a number of systemic options available, including gemcitabine (in combination with docetaxel or dacarbazine), trabectedin, and pazopanib. The National Comprehensive Cancer Network and the European Society for Medical Oncology do not define a set treatment sequence in advanced disease. Depending on availability, the sequence could be gemcitabine-based therapy followed by trabectedin and pazopanib. However, there have been no randomized comparative trials to inform the decision making process and the comparison of data between different randomized trials is exploratory and cannot be used for definitive recommendations. As a result, choosing between second-line options is to a certain extent arbitrary and can be guided by the relative advantages and disadvantages of each option in the context of each individual patient.

In DDLS, clear heterogeneity in tumor response to systemic therapy has been observed in a number of clinical trials. As of yet, no biomarkers are available which predict therapeutic response. However, in the context of rapidly progressive, symptomatic disease, a clear dose–response relationship of combination doxorubicin/ifosfamide has previously been demonstrated (11).

Well-differentiated liposarcoma and DDLS are frequently grouped together for histological subtype analysis in clinical trials. In up to 15% of patients, imaging can reveal concurrent areas of WDLS and DDLS within the same tumor mass (73). Is such cases, assessment of treatment response can be challenging as the predominantly fatty portions of WDLS are unlikely to exhibit any volumetric shrinkage and thereby underestimate treatment response by the DDLS component. As a result, there are limitations to available response criteria such as RECIST. Functional imaging may have a role in the future (74); however, as of yet they require further evaluation and validation in the context of WDLS and DDLS. Currently, the generalizability of trials grouping these patients remains questionable, due to the variable proportional representation of WDLS and DDLS in individual patients and their differing clinical response to treatment. More prospective data are required to answer these challenging questions.

The exciting developments as described in this study must, however, be tempered with the knowledge that histological specific evidence is scant still in this area but it does appear to be improving. Where it is available, the robustness of evidence is weakened by small patient numbers and subsequent difficulty with adequately powering of these studies. The difficulties enrolling patients with rare histological subtypes are a common theme across the spectrum of STS due to the heterogeneity of disease. Greater collaboration across specialist centers is imperative to improve the quality of research and subsequent evidence for subtype-driven management.

Given the recent developments in this area and the abundance of ongoing trials targeting multiple possible therapeutic pathways, the near future appears hopeful for the emergence of more definitive options and greater outcomes for patients with advanced disease.

## Author Contributions

YM and CZ wrote the first draft of the manuscript with input and guidance from RJ. All authors provided input into subsequent drafts.

## Conflict of Interest Statement

The authors declare that the research was conducted in the absence of any commercial or financial relationships that could be construed as a potential conflict of interest.
