# Supplementary material for: Systemic Therapy in Metastatic or Unresectable Well-Differentiated/Dedifferentiated Liposarcoma
Source: Front Oncol. 2017 Nov 30;7:292. doi: 10.3389/fonc.2017.00292 (PMC5715199; doi:10.3389/fonc.2017.00292)
Supplement: Supplementary file 1 [file Table_1.docx]

**Appendix 1**. Clinical trials investigating systemic therapies in WDLS/DDLS

| Study | Therapy | Phase | Population (n) | Reported endpoints | WDLS/DDLS analyses |
| --- | --- | --- | --- | --- | --- |
| Van Oosterom et al (2002) | Ifosfamide 5g/m^2^ over 24h every 3 weeks  VS  Ifosfamide 3g/m^2^ over 4h on 3 consecutive days every 3 weeks | II | Advanced STS  First line (103)  Second line (79) | First line:  RR 10% vs 25%  mPFS 11 vs 14 weeks  mOS 52 vs 44 weeks  Second line:  RR 6% vs 8%  mPFS 6 vs 14 weeks  mOS 45 vs 36 weeks | - |
| Judson et al (2014) | Doxorubicin (75mg/m^2^ intravenous bolus on day 1 or 72h infusion)  VS  Intensified doxorubicin (75mg/m^2^; 25mg/m^2^/d, days 1-3) plus ifosfamide (10g/m^2^ over 4 days) | III | Advanced STS (455) | RR 14% vs 26%***  mPFS 4.6 vs 7.4 months **  mOS 14.3 vs 12.8 months | - |
| Ryan et al (2016) | Doxorubicin (75mg/m^2^ day 1) plus palifosfamide (150mg/m^2^/d, days 1-3)  VS  Doxorubicin plus placebo | III | Advanced STS (447) | RR 28% vs 20%*  mPFS 6.0 vs 5.2 months  mOS 15.9 vs 16.9 months | - |
| Tap et al (2017) | Doxorubicin (75mg/m^2^ via bolus on day 1 or infusion)  VS  Doxorubicin plus evofosfamide (300mg/m^2^, day 1 and 8) | III | Advanced STS (640) | RR 18% vs 28%**  mPFS 6.2 vs 6.7 months  mOS 19.0 vs 18.4 months | - |
| Yovine et al (2004) | Trabectedin 1,500μg/m^2^ every 3 weeks | II | Advanced STS (54) | RR 4%  mPFS 1.9 months  mOS 12.8 months | - |
| Garcia-Carbonero et al (2004) | Trabectedin 1,500μg/m^2^ every 3 weeks | II | Advanced STS (36) | RR 8%  mPFS 1.7 months  mOS 12.1 months | - |
| Le Cesne et al (2005) | Trabectedin 1,500μg/m^2^ every 3 weeks | II | Advanced STS (104) | RR 8%  mPFS 105 days  mOS 9.2 months | - |
| Paz-Ares et al (2012) | Trabectedin 1,300-1650μg/m^2^ every 3 weeks plus dexamethasone (4mg every 12h for 7 doses) | II | Advanced STS (41) | RR 2%  mPFS 2.1 months  mOS 10.2 months | - |
| Demetri et al (2009) | Trabectedin 1,500μg/m^2^ every 3 weeks  VS  580 μg/m^2^ every week for 3 weeks of a 4 week cycle | II | Advanced liposarcoma and lipomyosarcoma (270) | RR 5.6% vs 1.6%  mPFS 3.3 vs 2.3 months*  mOS 13.9 vs 11.8 months | - |
| Bui-Nguyen et al (2015) | Doxorubicin 75mg/m^2^ infusion on day 1 every 3 weeks  VS  Trabectedin 1,300 μg/m^2^/3h infusion on day 1 every 3 weeks  VS  Trabectedin 1,500 μg/m^2^/24h infusion on day 1 every 3 weeks | IIb | Advanced STS (133) | RR 25.6% vs 14.8% vs 4.7%  mPFS 5.5 vs 2.8 vs 3.1 months | - |
| Blay et al (2014) | Doxorubicin 75mg/m^2^ plus ifosfamide 6-9g/m^2^ every 3 weeks  VS  Trabectedine 1,500 μg/m^2^/24h every 3 weeks | III | Translocation related sarcomas (121) | RR 27.0% vs 5.9%*  mPFS 8.3 vs 18.8 months | - |
| Demetri et al (2015) | Dacarbazine 1g/m^2^ every 3 weeks  VS  Trabectedine 1,500 μg/m^2^ every 3 weeks | III | Advanced liposarcoma and lipomyosarcoma (518) | RR 6.9% vs 9.9%  mPFS 1.5 vs 4.2 months***  mOS 12.9 vs 12.4 months | DDLS mPFS 1.9 vs 2.2 months |
| Le Cesne et al (2015) | Treatment interruption of trabectedin  VS  Continuation of trabectedin 1,500 μg/m^2^ every 3 weeks | II | Advanced STS with non-progressive disease following 6 cycles of trabectedin (91) | RR 3.6% vs 3.8%  mPFS 4.0 vs 7.2 months*  mOS 16.5 vs 27.9 months | - |
| Sessa et al (2009) | Trabectedin 600 μg/m^2^ plus doxorubicin 60mg/m^2^ every 3 weeks | I | Advanced STS (29) | RR 17.9% | - |
| Kasper et al (2015) | Trabectedin 700-900 μg/m^2^ on day 1 plus gemcitabine on day 1+8 every 3 weeks | I | Advanced liposarcoma and lipomyosarcoma (5) | RR 0% | - |
| Schöffski et al (2011) | Erubilin 1.4mg/m^2^ on day 1+8 every 3 weeks | II | Advanced liposarcoma (37) | RR 3%  mPFS 2.6 months | - |
| Schöffski et al (2016)  Chawla et al (2016) | Dacarbazine 850/1,000/1,200 mg/m^2^ every 3 weeks  VS  Erubilin 1.4mg/m^2^ on day 1+8 every 3 weeks | III | Advanced liposarcoma (153) | mOS 8.4 vs 15.6 months*** | DDLS mPFS 8.1 vs 18.0 months* |
| Patel et al (2001) | Gemcitabine 1g/m^2^ weekly | II | Advanced STS (56) | RR 18%  mPFS 3.0 months  mOS 13.9 months | - |
| Hartmann et al (2006) | Gemcitabine 1g/m^2^ on day 1+8+15 every 4 weeks | II | Advanced STS (19) | RR 7%  mPFS 3 months  mOS 6 months | - |
| Švancárová et al (2002) | Gemcitabine 1.25g/m^2^ on days 1+8 every 3 weeks | II | Advanced STS (32) | RR 3.2%  mPFS 45 days  mOS 268 days | - |
| Ferraresi et al (2008) | Gemcitabine 1g/m^2^ weekly for 3 weeks followed by 1 week rest | II | Advanced STS (14) | RR 7%  mPFS 3.1 months  mOS 11.8 months | - |
| Maki et al (2007) | Gemcitabine 1.2g/m^2^ on day 1+8 every 3 weeks  VS  Gemcitabine 0.9g/m^2^ on days 1+8 plus docetaxel 100mg/m^2^ on day 8 every 3 weeks | II | Advanced STS (122) | RR 8% vs 16%  mPFS 3.0 vs 6.2 months*  mOS 11.5 vs 17.9 months* | - |
| Buesa et al (1991) | Dacarbazine 1,200 mg/m^2^ every 3 weeks | II | Advanced STS (50) | RR 18% | - |
| Losa et al (2007) | Gemcitabine 1,800 mg/m^2^ plus dacarbazine 500mg/m^2^ every 2 weeks | II | Advanced STS (26) | RR 4%  mPFS 15.4 weeks  mOS 37 weeks | - |
| Dickson et al (2013) | Palbociclib 200mg for 14 days every 3 weeks | II | WDLS/DDLS (30) | - | RR 3%  mPFS 17.9 weeks |
| Dickson et al (2016) | Palbociclib 125mg for 21 days every 4 weeks | II | WDLS/DDLS (60) | - | mPFS 17.9 weeks |
| Luke et al (2012) | Doxorubicin 60-75 mg/m^2^ on day 1 plus flavopiridol 40-70 mg/m^2^ on day 1 every 3 weeks | I | Advanced STS (31) | RR 6% | - |
| Mahmood et al (2011) | Sunitinib 50mg for 28 days every 6 weeks | II | Advanced liposarcoma (18) | RR 0%  mPFS 3.4 months  mOS 16.9 months | - |
| Von Mehren et al (2012) | Sorafenib 400mg twice daily for 28 days | II | Advanced STS (51) | RR 0%  mPFS 3 months  mOS 17 months | - |
| Sleijfer et al (2009) | Pazopanib 800mg daily | II | Advanced liposarcoma (19) | mPFS 80 days  mOS 197 days | - |
| Van der Graaf et al (2012) | Pazopanib 800mg daily VS  Placebo | III | Advanced STS (372) | RR 6%  mPFS 4.6 months***  mOS 12.5 months | - |
| Tap et al (2016) | Doxorubicin 75mg/m^2^ on day 1 every 3 weeks  VS  Olaratumab 15mg/kg on day 1+8 plus doxorubicin 75mg/m^2^ on day 1 every 3 weeks | Ib/II | Advanced STS (133) | RR 11% vs 18%  mPFS 4.1 vs 6.6 months  mOS 14.7 vs 26.5 months *** | - |
| Chawla et al (2015) | Doxorubicin 75/m^2^ on day 1 every 3 weeks  VS  Aldoxorubicin 350mg/m^2^ on day 1 every 3 weeks | IIb | Advanced STS (126) | RR 25% vs 0%  mPFS 2.7 vs 5.6 months*  mOS 14.3 vs 15.8 months | - |
| Chawla et al (2017) | Investigators choice  VS  Aldoxorubicin 350mg/m^2^ on day 1 every 3 weeks | III | Advanced liposarcoma and lipomyosarcoma (249) | mPFS 3.0 vs 5.3 months** | - |
| Burgess et al (2017) | Pembrolizumab 200mg every 3 weeks | II | Advanced STS and bone sarcoma (86) | RR 18% | DDLS 20% |
| Gounder et al | Selinexor 30-50mg/m^2^ or 60mg on day1+3 every week | Ib | Advanced STS and bone sarcoma (54) | RR 0% | - |

RR – Response rates

mPFS – Median progression free survival

mOS – Median overall survival

* Statistically significant p<0.05

** Statistically significant p<0.01

*** Statistically significant p<0.001
